# Are there benefits of culture-based detection of *Mycobacterium avium* spp paratuberculosis over histopathology?

**DOI:** 10.4102/ojvr.v92i1.2159

**Published:** 2025-02-11

**Authors:** Motlatso T. Hlokwe, Nomawethu S. Masina, Boitumelo Letsoko, Sewellyn C. Davey, Anita L. Michel

**Affiliations:** 1 Tuberculosis Laboratory, Diagnostic Services Programme, ARC-Onderstepoort Veterinary Research, Pretoria, South Africa; 2 Department of Veterinary Tropical Diseases, Bovine TB and Brucellosis Research Programme, Faculty of Veterinary Sciences, University of Pretoria, Pretoria, South Africa; 3 Tuberculosis Laboratory, Diagnostic Services Programme, ARC-Onderstepoort Veterinary Research, Onderstepoort, Pretoria, South Africa; 4 Mammalian Serology Laboratory, Potchefstroom Provincial Veterinary Laboratory, Potchefstroom, South Africa; 5 Department of Agriculture, Veterinary Services, Animal Health, State Veterinarian Malmesbury, Malmesbury, South Africa

**Keywords:** *Mycobacterium avium* subspecies, paratuberculosis, Ovine Johne’s Disease, VersaTREK automated liquid culture system, antibiotic cocktail

## Abstract

Paratuberculosis (Johne’s disease) has devastating outcomes on ruminant health and impacts on national and international trade. The current work assessed the diagnostic value of the VersaTREK automated liquid culture system in isolating *Mycobacterium avium* subspecies paratuberculosis (MAP) from faecal and intestinal tissue samples from ovine under South African conditions and compared it with the current method of choice, histopathological examination. Intestinal tissue and faecal samples from 111 sheep (including complete set from 104 slaughter sheep from flocks with a history of MAP infection as well as incomplete sample sets from 7 sheep) were analysed using the liquid culture method. One set of tissues was subjected to histopathological examination. Deoxyribonucleic acid (DNA) extracted from culture isolates was subjected to polymerase chain reaction (PCR) amplification using primers that target the IS900 regions of the MAP for species verification. Overall, the VersaTREK automated liquid culture in combination with IS900 PCR showed a comparable level of detection in tissues (12.6%) as histopathology (13.5%), but the detection rate for faecal samples was lower than for tissues (10.8%). A combination of histopathology and faecal culture increased the detection rate from 13.5% (*n* = 14/104) and 9.6% (*n* = 10/104), respectively, to 15.4% (*n* = 16/104).

**Contribution:**

Our findings highlight the diagnostic utility of the VersaTREK automated liquid culture system in detecting MAP in ovine samples collected both ante and postmortem. However, an inhibitory effect on the MAP isolation rate observed when the antibiotic cocktail was added to the culture medium warrants further investigation. The outcome of the study is beneficial in guiding the strategic planning of the nationwide control programme.

## Introduction

Paratuberculosis (Johne’s disease) poses a big threat to ruminant livestock and has a significant effect on the economy (Motiwala et al. 2004); hence, one of the reasons it should be controlled (Whittington et al. 2019). The disease has devastating outcomes on animal health and impacts on national and international trade, and remains endemic in many parts of the world (Gao et al. 2002). In addition, animals with unknown paratuberculosis status slaughtered at abattoirs pose a high risk of disseminating the causative organism in the abattoir environment and may contaminate other meat being processed, posing a risk for human consumption (Gao et al. 2002). The causative agent of Johne’s disease is *Mycobacterium avium* subspecies *paratuberculosis* (MAP), which belongs to genus *Mycobacterium* (Thorel, Blom-Potar & Rastog 1990). Because the incubation period of paratuberculosis in an animal can vary from months to several years, the disease is not manifested clinically until the animal becomes an adult, but the majority may remain sub-clinical carriers (Whittington & Windsor 2009). The disease is also commonly reported in wild ruminant species in several countries (Thomas et al. 2021) and was diagnosed in a domestic dog from the Western Cape province in South Africa; the source was suspected to be infected sheep in the nearby grazing area (Miller et al. 2017). There is no effective treatment for paratuberculosis, and clinically affected animals ultimately die from the disease (Whittington 2009).

The disease in ovine flocks has been increasingly reported in countries such as Australia, South America, South Africa, among others (Masina 2018; Windsor 2014). In South Africa, the distribution and prevalence of the disease remain poorly investigated, and the veterinary scientific community is still far from reaching consensus of a better way to deal with it. Currently, there are indications that paratuberculosis cases are on the rise. For many years, MAP was only diagnosed serologically in the country, but the downside of such tests is that they have low sensitivity and are costly (Whittington et al. 2000). The causative agent can only be confirmed by culture; hence, the main aim of the current study was to conduct a comparative evaluation of the diagnostic performance of the VersaTREK automated liquid culture system for the isolation of MAP from sheep faecal and tissue samples, compared with the most used method in South Africa, histopathology.

## Research methods and design

### Study areas

For this study, a convenience sampling strategy was adopted, whereby sheep from selected flocks with suspected paratuberculosis positive status from the Western Cape, Eastern Cape, and Free State were sampled at routine slaughter. Animal slaughter was conducted at local abattoirs. As negative controls, faecal samples from a total of 50 sheep from 9 different sheep flocks from Gauteng province with no previous history of a positive paratuberculosis diagnosis were collected.

### Sample collection

Sample collection was done by the responsible State Veterinarian with the assistance of qualified Animal Health Technicians between the years 2014 and 2018. During collection, complete sample sets consisting of duplicate tissue samples (pooled ileocecal valve area and mesenteric lymph node) and faecal samples were collected from 104 slaughter sheep as well as incomplete sample sets from 7 sheep (allowing partial analyses for these animals) from flocks with a known history of MAP infection in the flock. A set of tissues was fixed in buffered formalin and instantly dispatched for histopathological examination. The other group of tissues and corresponding faeces were chilled in cooler box with ice packs and consigned to the laboratory. Isolation of MAP was conducted at the Agricultural Research Council-Onderstepoort Veterinary Research (ARC-OVR) using the VersaTREK automated liquid culture system. In addition, faecal samples (*n* = 50) were collected from different randomly selected smallholdings in Gauteng regions which were known to be free of paratuberculosis due to their flock histories being negative for MAP. All samples were dispatched cold to the laboratory, where they were frozen at –20 °С until required (Masina 2018).

### Sample identification

Collected tissue and faecal samples were dispatched along with submission forms which contained further details of the sheep sampled, farmer and exact locality of small holding, and unique identification number. Upon dispatch at the ARC-OVR’s Tuberculosis laboratory, samples were allocated identification numbers (sequential) named ‘Paratuberculosis (PTB) numbers’, or ‘Validation Paratuberculosis’ (VPTB) numbers (Masima 2018).

### Sample processing

#### Processing of faecal samples

On the day of processing, samples were left at ambient temperature to thaw. The Cornell double incubation decontamination method for MAP isolation from faeces was used (Kim et al. 2004). Approximately 2.0 g – 2.5 g of the faecal sample from individual animals was homogenised in 35 mL of sterile distilled water in a 50 mL centrifuge tube prefilled with 35 mL of sterile H_2_O, followed by agitation to disrupt the faecal pellets (Masina 2018). Solid particles in the homogenised sample were allowed to sediment by incubation at ambient temperature (18 °C – 26 °C) for 30 min to 60 min. Five millilitres of the supernatant was aliquoted into a 25 mL of 0.9% cetylpyridinium chloride monohydrate (CPC, final concentration 0.75%). The mixture was incubated at 37 °C for 18 h to 24 h as previously described (Kim et al. 2004). Following incubation, the blend was centrifuged (Allergra^TM^ X-22R centrifuge, Beckman Coulter Inc, California) for 20 min at 3500 rpm (revolutions per minute) at ambient temperature for pellet collection. The supernatant was discarded, and 1 mL of an antibiotic cocktail containing vancomycin, amphotericin B and nalidixic acid (ESP para-JEM AS) was added to re-suspend the pellet. The sample was incubated for 18 h to 24 h at 37 °C before proceeding to culture.

#### Processing of tissue samples

During collection, sheep intestinal tissue samples from the ileocecal valve area, ileum, ileocecal and mesenteric lymph nodes were pooled before dispatch to the laboratory. Fat was trimmed from thawed tissue samples using sterile pair of scissors and sliced into tiny pieces. Thirty-five millilitres of distilled sterile was used to homogenise the tissue pool with a blade homogeniser. The homogenate was sedimented at ambient temperature for 30 min to 60 min. Five millilitres of the supernatant was carefully drawn using a pipette without disturbing the sediments and was transferred into 25 mL of 0.9% CPC. The mixture was incubated at 37 °C overnight (18 h – 24 h). Further processing was conducted as described for the faecal samples. The remaining sample was stored in a –20 °C freezer.

#### Mycobacterial culture of tissue and faecal samples

Intestinal tissue and faecal samples from 111 sheep (including complete set from 104 slaughter sheep as well as incomplete sample sets from 7 sheep) were cultured according to Kim et al. (2004). One millilitre of each ESP para-JEM growth supplement and ESP para-JEM egg yolk supplement, 500 µL of ESP para-JEM antibiotic supplement and 50 µL of para-JEM Blue were inoculated into a specialised VersaTREK ParaJEM bottle containing 11 mL of culture broth. The decontaminated samples were then inoculated individually into the VersaTREK ParaJEM bottles and blended thoroughly to ensure effective suspension of nutrients and sample. Culture bottles were loaded on the VersaTREK automated liquid culture system according to manufacturer’s instructions and incubated for a maximum of 70 days.

#### Microscopy

Culture bottles indicating a positive signal on the VersaTREK automated liquid culture system were removed from the system. Smears were prepared from the centrifuged culture pellet, and Ziehl Neelsen staining and microscopic examination were performed to examine acid fastness of the cultures as previously described (Shinnick & Good 1995).

#### Preparation of deoxyribonucleic acid template

Culture samples detected as positive by the VersaTREK automated liquid culture system, and those identified as negative following the completion of the incubation period were subjected to deoxyribonucleic acid (DNA) extraction and subsequent polymerase chain reaction (PCR) amplification. Two millilitres of each of the cultures were transferred into individually labelled 2 mL centrifuge tubes and centrifuged for 10 min at 3500 rpm for pellet harvesting. Deoxyribonucleic acid extraction was done automatically using the Maxwell 16 system (Anatech instruments (Pty) Ltd; www://anatec.co.za) following manufacturer’s instructions, or by using the boiling method (suspension of the pellet in 100 mL sterile distilled water and boiling for 20 min). The extracted DNA was chilled (2 °C – 8 °C) until PCR test was conducted.

#### *Mycobacterium avium* subspecies paratuberculosis species verification

Extracted DNA was subjected to amplification for MAP identification using oligonucleotides (primers) targeting the IS900 sequence region of the MAP genome (Kim et al. 2002; Moghadam et al. 2010). Primer (Whitehead Scientific, [Pty] Ltd, South Africa) sequences used were as follows: IS900 (P90) Forward: 5’-GAAGGGTGTTCGGGGCCGTC-3’; IS900 (P91) Reverse: 5’-GAGGTCGATCGCCCACGTGA-3’. Deoxyribonucleic acid amplification was carried out in a 50 µL reaction consisting of 10 µL of template DNA, 29 µL of DNA-free H_2_O and 1 µL of each 20 mM primer, 2 µL dNTP, 3 µL MgCl_2_, 5 µL 10x buffer and 0.25 µL HotStar Taq polymerase. Polymerase chain reaction was carried out in a thermocycler (Eppendorf AG 22331 Hamburg, Germany). The DNA amplification conditions included: initial denaturation at 95 °C for 10 min and subsequent 50 cycles of denaturation at 95 °C for 1 min, annealing at 65 °C for 1 min, extension at 72 °C for 1 min and a final extension step at 72 °C for 10 min (Masina 2018). To visualise DNA, the amplicons were separated using agarose gel electrophoresis and analysed under ultraviolet (UV) light. The resulting DNA fragment band sizes were estimated by comparison with a size marker (A 100 base pairs [bp] DNA ladder, Fermentas Life Sciences, Vilnus, Lithuania).

### Evaluation of the analytical specificity of the IS900 *Mycobacterium avium* subspecies paratuberculosis polymerase chain reaction test

To evaluate the analytical specificity of the IS900 PCR method used for MAP species verification, a panel of *Mycobacterium* species including members of the *Mycobacterium tuberculosis* complex (i.e., *Mycobacterium bovis, n* = 2; *Mycobacterium tuberculosis, n* = 4; *Mycobacterium orygis, n* = 2 and *Mycobacterium suricattae, n* = 1) as well as non-tuberculous mycobacteria (NTM) (*Mycobacterium fortuitum* ATCC 6841, *n* = 1 and unidentified NTM, *n* = 3) were analysed by PCR using primers targeting the IS900 insertion sequences as described in previous reports (Kim et al. 2002; Moghadam et al. 2010). Analysis of the samples was carried out by two different analysts.

### Histopathological examination of tissue samples

Formalin (10%) fixed intestinal tissue samples were dispatched instantly to either one of the three laboratories, that is, the Western Cape Provincial Veterinary Laboratory (WCPVL), the Vet Diagnostix Laboratory (Cape Town) or Pathcare Laboratory (Cape Town) for histopathology analysis and diagnosis. Tissue samples from 104 samples were analysed histopathologically. Samples were cut in, embedded and processed to wax blocks using routine methods for histopathology. They were evaluated for thickened intestinal walls infiltrated by plasma cells, epithelioid macrophages and lymphocytes resulting from the immune response to MAP infection. Ziehl Neelsen staining to visualise MAP in the tissue sections served as confirmation. Results of the histopathological examination, tissue and faecal culture were evaluated comparatively.

### Culture of ovine faecal and intestinal tissue samples with and without antibiotic cocktail

Thirteen pairs of tissue and faecal samples as well as three individual tissue samples were cultured with and without the recommended antibiotics cocktail in the VersaTREK medium. These samples were selected based on the following criteria: (1) a known MAP positive tissue sample as confirmed by either culture, PCR or histopathology method but tested negative on at least one of these test procedures; (2) one of two paired samples that tested positive on at least one test method. Samples were decontaminated as previously described (Kim et al. 2004). Culturing was conducted in duplicates for individual samples, with or without the addition of an antibiotic supplement (culture bottles labelled A and B, respectively).

### Statistical analysis

The correlation between faecal and tissue sample culture, and histopathology was assessed from the complete set of samples (*n* = 104) using Pearson’s correlation coefficient. Coefficient values between ± 0.50 and ± 1 were interpreted as a strong correlation, between ± 0.30 and ± 0.49 as medium correlation and values below + 0.29 as low correlation (Tylor 1999). The agreement between the culture methods and histopathology was assessed using Cohen’s kappa based on the complete sample set (*n* = 104) and the additional incomplete set was included for the agreement between the culture methods (*n* = 111). All statistical procedures were performed using IBM^®^ Statistical Package for Social Sciences (SPSS) Statistics (Version 26, International Business Machines Corp., Armonk, New York, United States) and results were interpreted at the 5% level of significance.

### Ethical considerations

Ethical approval to conduct this study was obtained from the Department of Agriculture, Land Reforms and Rural Development (DALRRD) (reference number: 12/11/1/1). The ARC-OVR Onderstepoort Veterinary Institute Animal Ethics Committee (reference number: AEC 13.16) and University of Pretoria Animal Ethics Committee (Project number: V142-16) Animal Ethics Committees.

## Results

### Faecal and tissue culture

**FIGURE 1:**
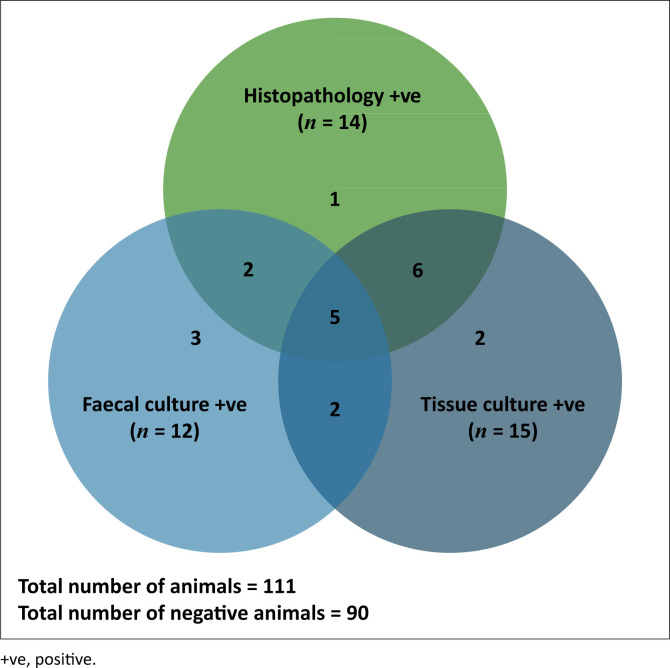
Summary of paratuberculosis positive sheep identified by faecal culture, and/or tissue culture, and/or histopathology based on the complete sample set and the additional incomplete set between the culture methods (*N* = 111).

Tissue samples from 15 (13.5%) and faecal samples from 8 (7.2%) out of 111 sheep indicated positive growth signals from the VersaTREK automated liquid culture system following incubation at 37 °C (Figure 1 & Table 1-A1). Culture bottles that yielded no positive growth signals were declared negative after 70 days (normal set incubation time) and their content was further processed for PCR. For samples yielding a positive signal the time to detection (TTD) ranged from 6–43 days for tissue samples, and 6–56 days for faecal samples, with an additional isolation made from one of the faecal samples after the set time of incubation (79 days). Deoxyribonucleic acid extraction for PCR analysis was carried out for all but one cultured sample.

### Microscopy

Acid fast rods typical of MAP were observed microscopically for all samples that gave a positive signal on the VersaTREK automated liquid culture system.

### Identification of *Mycobacterium avium* subspecies paratuberculosis by polymerase chain reaction (species verification)

Fifteen tissue culture and eight faecal culture samples yielded a positive growth signals in the automated liquid culture system and were subjected to PCR speciation. Deoxyribonucleic acid extracted from 14 tissue and 7 faecal samples amplified the IS900 region (400 bp product) of the MAP genome by conventional PCR. Additionally, five faecal cultures with negative growth results were found to be PCR positive for MAP (Figure 1 & Table 1-A1). The corresponding tissue culture was positive for one of the sheep, and two sheep were positive by histopathology. The final tissue and/or faecal culture outcome was based on verification by PCR amplification; hence, the detection rate of MAP in tissue samples was 14 out of 111 (12.6%), 10 out of 104 (9.6%) and in faecal samples 12 out of 111 (10.8%) and 10 out of 104 (9.6%).

### Evaluation of the analytical specificity of the IS900 *Mycobacterium avium* subspecies paratuberculosis polymerase chain reaction test

**FIGURE 2:**
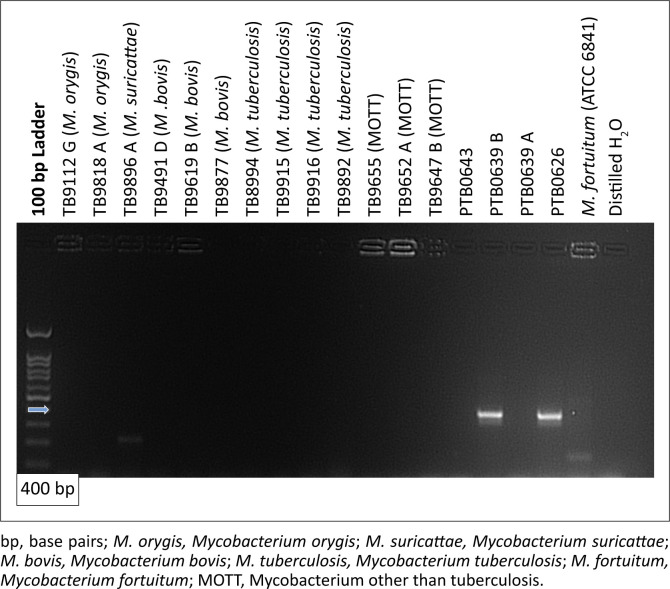
IS900 polymerase chain reaction gel electrophoresis results for the determination of analytical specificity. A 100 bp deoxyribonucleic acid ladder was used to estimate the polymerase chain reaction product sizes. *Mycobacterium* species analysed included a variety of *Mycobacterium tuberculosis* complex strains, *Mycobacterium avium* subspecies paratuberculosis isolates and non-tuberculous *Mycobacterium* strains. An expected polymerase chain reaction product size of 400 bp was observed only in *Mycobacterium avium* subspecies paratuberculosis positive isolates (i.e., PTB0639 A and PTB0626). MOTT indicates *Mycobacterium* other than tuberculosis. Sterile distilled water was used as a blank.

As indicated in Figure 2, only isolates previously identified as MAP isolates (i.e., PTB 0355 and VPTB 116) resulted in amplification of the IS900 region by producing the expected 400 bp PCR product size. None of the 50 faecal samples from sheep originating from MAP free flocks were PCR positive for MAP, neither did any of the 10 *Mycobacterium tuberculosis* complex species and 4 NTM yield a PCR product. The same test results were obtained by both analysts.

### Histopathology

Of the 104 animals analysed on histopathology, 14 were diagnosed positive (13.5%) for paratuberculosis (Johne’s disease) (Figure 1 & Table 1-A1) because of the presence of thickened intestinal walls infiltrated by epithelioid macrophages, plasma cells and lymphocytes as a result of immune system’s response to MAP infection and acid fast rods typical of MAP. In some cases, acid fast bacteria were not observed on histopathology when subjected to Ziehl Neelsen staining; however, a positive diagnosis was made based on lesions that were pathognomonic for paratuberculosis. A total of 90 sheep were diagnosed as negative for paratuberculosis by histopathology.

### Sensitivity and parallel interpretation of histopathology and culture results

A total of 21 sheep out of the 104 sheep were classified as paratuberculosis positive by one or more of the test methods (Figure 1 & Table 1-A1). Histopathology detected 14 out of 17 tested (80.9%), faecal culture (plus PCR confirmation) 12 out of 19 (63.1%) and tissue culture (plus PCR confirmation) 14 out of 21 (66.1%). Parallel interpretation (an animal is considered positive if at least one of several tests conducted gives a positive result) of tissue culture and faecal culture and of histopathology and faecal culture increased the sensitivity in both approaches to 19 out of 19 tested (100%), while the combined sensitivity of histopathology and tissue culture remained at 80.9% (*n* = 14/17). The detection rate of MAP among 111 animals (incomplete sets) from infected flocks increased from 12.6% and 10.8%, respectively, to 17.1% (*n* = 19/111) for parallel interpretation of tissue and faecal culture. For histopathology and faecal culture applied to 104 sheep (complete set), the detection rate increased from 13.5% (*n* = 14/104) and 9.6% (*n* = 10/104), respectively, to 15.4% (*n* = 16/104). When histopathology was combined with faecal and tissue culture results, the detection rate remained at 13.5%.

### Correlation between histopathology and faecal culture and tissue culture

There was a significant, large positive relationship between tissue culture and histopathology (Pearson’s correlation coefficient *r* = 0.85) (*p* < 0.001) and between faecal culture and histopathology (*r* = 0.59) (*p* < 0.001). A significant, but lower, positive relationship was found between tissue and faecal culture (*r* = 0.363) (*p* < 0.001). There was an almost perfect test agreement of 97.1% (Cohen’s kappa: *k* = 0.86) between tissue culture and histopathology, a substantial agreement of 93.3% (Cohen’s kappa: *k* = 0.63) between histopathology and faecal culture and a moderate agreement of 88.3% (Cohen’s kappa: *k* = 0.41) for faecal and tissue culture.

### Effect of the antibiotic cocktail on the recovery of *Mycobacterium avium* subspecies paratuberculosis strains from infected ovine tissue and faecal samples

**FIGURE 3:**
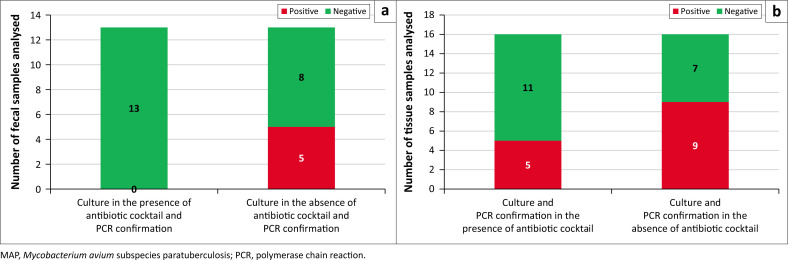
Culture of *Mycobacterium avium* subspecies paratuberculosis from ovine faecal (a) and tissue (b) samples in the presence or absence of an antibiotic cocktail supplement. Green and red bars represent negative and positive samples respectively.

A separate experiment was carried out to determine if antibiotic supplementation of the VersaTREK medium was necessary after CPC decontamination. In the presence of the antibiotic cocktail recommended by the manufacturer, MAP was isolated from 5 (31.25%) out of 16 tissue samples cultured. The isolation rate doubled (*n* = 10/16, 62.5%) when tissues were cultured without this antibiotic cocktail. No MAP isolation was made from faecal samples processed and cultured in a medium containing the antibiotic cocktail. However, MAP was isolated from 5 of the 13 samples processed and cultured in the absence of an antibiotic cocktail supplement (Figure 3 & Table 2-A1). No contamination was detected for faecal and intestinal tissues cultured in the absence of the antibiotic cocktail; therefore, CPC decontamination process had been 100% effective in inactivating non-target microorganisms.

## Discussion

In 1967, an imported merino ram was diagnosed with Johne’s disease in South Africa, which marked the initial reported case of the disease in the country (Van Niekerk & Van der Walt 1967). Currently, there are indications that paratuberculosis cases are on the rise in sheep flocks and cattle herds in the country. According to the Department of Agriculture, Land Reforms and Rural Development (DALRRD), the disease was reported in all provinces between the years 1993 and 2022, and more cases were reported in sheep in the Western Cape Province (G. de Klerk, pers. comm., 02 August 2023). Officially, paratuberculosis is a controlled disease in South Africa, supported by a national control scheme, but control measures are limited to quarantine of the affected flock or herd and the registration of the Gudair^®^ vaccine for use in sheep (Department of Agriculture, Forestry and Fisheries, Republic of South Africa 2017). Confirmation of MAP infection by culture is the gold standard (Sohal et al. 2007) but is complicated, especially in sheep, by the chronic nature of the disease, the poor cultivability of sheep strains of MAP as well as the intermittent excretion of MAP (Whittington 2009). In low- and middle-income countries like South Africa, paratuberculosis is commonly diagnosed by histopathology although this accepted technique cannot in all cases provide a confirmed diagnosis (Whittington 2009). In view of the ongoing spread of paratuberculosis throughout South Africa, a culture-based confirmatory diagnostic approach is not only an advantage but essential to support strategic national and industry-driven disease control measures. Histopathological tissue examination is a well-established method with a short turnaround time of several days as compared to several weeks for culture-based diagnosis. Formalin-fixed tissue samples are cost-effective to collect and ship to a laboratory. On the downside, histopathology is limited to postmortem samples, depends on the availability of highly trained veterinary pathologists, and does not in all cases allow a confirmed diagnosis.

The VersaTREK automated liquid culture system has been used internationally for routine MAP testing (Okwumabua et al. 2010; Van Maanen et al. 2000); hence, our main objective was to compare this system for culture of intestinal tissue and faecal samples with histopathology under South African conditions. In this study, the VersaTREK automated culture system in combination with conventional PCR targeting the IS900 region of MAP showed a 100% specificity among faecal samples from uninfected sheep. To further explore the specificity of the primers used in this study, a variety of *M. tuberculosis* complex strains as well as selected NTM were analysed. None of these strains amplified the IS900 region, supporting the diagnostic use of the IS900 PCR in the MAP species verification.

In sheep from MAP infected flocks, it was shown that culture was both most specific and sensitive when it was applied in combination with confirmatory IS900 PCR as this allowed the positive diagnosis of MAP in faecal cultures from five sheep which indicated a negative result on the VersaTREK automated liquid culture system (Figure 1 & Table 1-A1). In three of these animals, infection was confirmed either by culture of the tissues (2017-D-624) or by histopathology (2017-D-2277-1&2).

Cultivability of the circulating faecal MAP strain was questioned in these latter cases, as it could be possible that the antibiotic cocktail was not conducive for MAP growth in the early stages. A similar observation was made in the United States (US) in a study conducted by Ellingson, Koziczkowski and Anderson (2004), where additional faecal samples tested PCR positive with IS900 primers, although culture negative by VersaTREK and Herrold’s egg yolk agar (HEYA) culture systems. In conclusion, negative cultures (no growth signals detected) should, as a rule, be tested by PCR at the end of incubation. This should, however, be done taking into consideration the total cost of testing involved.

Faecal culture detected MAP in 10.8% of animals which was slightly less than histopathology (13.5%) and culture of tissues (12.6%). When used in parallel with histopathology, the detection rate of faecal culture increased to 15.4% (*n* = 16/104) and when combined with tissue culture to 17.1% in the total sample size of 111 sheep. This suggests an improved diagnostic performance from testing both faecal and tissue samples in parallel, whereby both tissue culture and histopathology seemed to add comparable benefits to faecal culture. Since this approach will increase the costs for diagnosis, it should be recommended only when maximum sensitivity is required for example, in the case of confirming suspected infection in a previously negative flock.

Faecal culture offers the additional advantage of an ante mortem diagnostic tool in the monitoring the MAP infection status in flocks and herds. As a further saving, costs can be reduced by pooling of faecal samples from a cohort of sheep (Whittington et al. 2000).

Overall, the strong and/or medium positive correlation between tissue culture and histopathology and between faecal culture and histopathology (Pearson’s *r* = 0.85 and *r* = 0.59, respectively) confirms that the tests produce comparable results. The lower positive correlation (*r* = 0.36) between faecal culture and tissue culture may suggest that faecal shedding was not strongly associated with the bacterial load in intestinal tissues, in which case a parallel use of the methods could increase the diagnostic performance. It may also suggest that the sensitivity of faecal culture was compromised by the presence of antibiotics in the culture medium. The strong and/or substantial test agreement of histopathology with tissue and faecal culture, respectively, confirmed that the methods were highly comparable.

As shown in this study, it is important to take into consideration, the slow growth of MAP, particularly sheep strains, in the optimisation of the method. One of the faecal samples took beyond the set incubation period (70 days) to give a positive signal only after 79 days, emphasising the importance of local validation of a test method. Nevertheless, and although sheep MAP strains are known to be difficult to isolate compared to cattle strains, the system was able to detect MAP as early as 6 days in culture, thus offering detection potentially as early as histopathology for samples with high MAP load.

Antibiotics have previously been reported to have a detrimental effect on the growth of MAP resulting in a reduced number of viable cells in the culture, and a reduced sensitivity of culture-based tests (Biet & Boschiroli 2014; Bradner et al. 2013). In this study, we conducted a pilot evaluation of samples cultured in a medium containing the recommended antibiotic cocktail for the VersaTREK automated liquid culture system, and without this supplement. Our results showed approximately 50% reduction in the isolation of MAP from tissue samples processed and cultured in the presence of antibiotic cocktail supplement. The adverse effect of the supplement appeared to be dire in faecal samples, with no (0%) isolation made. Furthermore, we noted that time to detection (TTD) was greatly reduced by up to 75% in some of the tissue samples cultured without an antibiotic cocktail (i.e., from isolation in 20 days to only 5 days), whereas MAP was isolated within 5 days and 12 days in faecal samples processed and cultured without an antibiotic supplement. Although a limited sample size was analysed, our findings suggest that the antibiotic cocktail may have contributed to the low detection rate of the faecal culture observed in this pilot study and potentially also in the field study. Decontamination with CPC alone did provide for adequate recovery of viable MAP from tissue and faecal samples in this study. It is, however, recommended that further optimisation including evaluation of the detection limit of the method, be conducted.

## Conclusion

Histopathology detected slightly more positive animals than the VersaTREK liquid culture system, in agreement with previous reports (Coelho et al. 2017; Younus et al. 2012) but was closely followed by tissue culture. Parallel interpretation of tissue culture with faecal culture provided a more sensitive diagnostic approach compared to histopathology alone in this study. The study recommends further work to be conducted regarding the effect of the recommended antibiotic cocktail supplement. Overall, our findings support the use of VersaTREK automated liquid culture system in detecting viable MAP in samples from infected sheep flocks. The culture system will be instrumental in increasing the much-needed diagnostic capacity together with histopathology and provide adequate support to the paratuberculosis control programme in South Africa.

## Appendix 1

**TABLE 1-A1:** Paratuberculosis positive sheep identified by faecal culture, and/or tissue culture, and/or histopathology based on the complete sample set and the additional incomplete set between the culture methods (*n* = 111).

Sample identification	Test results per type of test		
	Histopathology	Faecal culture	Tissue culture
2017-D-19084	ND	+ ve (Signal, ZN, PCR)	+ ve (Signal, ZN, PCR)
2017-D-824	+ ve	+ ve (Signal, ZN, PCR)	+ ve (Signal, ZN, PCR not done)
2017-D-826	+ ve	- ve	+ ve (Signal, ZN, PCR)
2016-D-21453	- ve	+ ve (Signal, ZN, PCR)	- ve
2014-D-18090	ND	- ve	+ ve (Signal, ZN, PCR)
2014-D-11501	+ ve	ND	+ ve (Signal, ZN, PCR)
2017-D-3160	+ ve	+ ve (Signal, ZN, PCR)	+ ve (Signal, ZN, PCR)
2016-D-17706	+ ve	- ve	+ ve (Signal, ZN, PCR)
2017-D-3159	+ ve	- ve	+ ve (Signal, ZN, PCR)
2017-D-1101	+ ve	- ve	+ ve (Signal, ZN, PCR)
2017-D-1440	+ ve	+ ve (No signal but PCR positive)	- ve
2017-D-2277 (1)	+ ve	- ve	+ ve (Signal, ZN, PCR)
2016-D-10251	ND	- ve	+ ve (Signal, ZN, PCR)
2017-D-624	ND	+ ve (No signal but PCR positive)	+ ve (Signal, ZN, PCR)
2017-D-2277 (2)	+ ve	+ ve (No signal but PCR positive)	- ve
2015-D-20205 (VPTB4)	- ve	+ ve (No signal but PCR positive)	- ve
2015-D-20205 (VPTB35)	- ve	+ ve (No signal but PCR positive)	- ve
2016-D-6124	+ ve	ND	- ve
2018-D-770 (VPTB263)	+ ve	+ ve (Signal, ZN, PCR)	+ ve (Signal, ZN, PCR)
2018-D-770 (VPTB264)	+ ve	+ ve (Signal, ZN, PCR)	+ ve (Signal, ZN, PCR)
2018-D-770 (VPTB265)	+ ve	+ ve (Signal, ZN, PCR)	+ ve (Signal, ZN, PCR)

+ve, positive result obtained; -ve, negative result obtained; ND, not done; ZN, Ziehl Neelsen staining; PCR, polymerase chain reaction

**TABLE 2-A1:** Culture of ovine tissue and faecal samples in the presence or absence of an antibiotic cocktail supplement.

Sample identification Tissue (B)	Antibiotic present (N) cultured results & signal/(TTD)	PCR confirmation (N)	Antibiotic absent (NA) cultured signal B/(TTD)	PCR confirmation NA	Sample identification Faecal (A)	Antibiotic present (N) cultured results & signal (TTD)	PCR confirmation (N)	Antibiotic absent (NA) cultured signal A/(TTD)	PCR confirmation NA
VPTB57B	Negative	Negative	Positive (6 days)	Positive	VPTB75A	Negative	Negative	Negative	Negative
VPTB60B	Negative	Negative	Negative	Negative	VPTB105A	Negative	Negative	Negative	Negative
VPTB61B	Negative	Negative	Positive (12 days)	Positive	VPTB106A	Negative	Negative	Positive (3 days)	Negative
VPTB75B	Negative	Negative	Negative	Negative	VPTB108A	Negative	Negative	Negative	Negative
VPTB105B	Negative	Negative	Negative	Negative	VPTB109A	Negative	Negative	Negative	Negative
VPTB106B	Negative	Negative	Negative	Negative	VPTB110A	Negative	Negative	Negative	Negative
VPTB108B	Negative	Negative	Negative	Negative	VPTB111A	Negative	Negative	Negative	Negative
VPTB109B	Negative	Negative	Positive (5 days)	Negative	VPTB112A	Negative	Negative	Positive (5 days)	Positive
VPTB110B	Negative	Negative	Positive (28 days)	Positive	VPTB113A	Negative	Negative	Negative	Negative
VPTB111B	Positive (25 days)	Positive	Positive (12 days)	Positive	VPTB114A	Negative	Negative	Positive (5 days)	Positive
VPTB112B	Negative	Negative	Positive (5 days)	Positive	VPTB115A	Negative	Negative	Positive (12 days)	Positive
VPTB113B	Positive (21 days)	Positive	Positive (12 days)	Positive	VPTB116A	Negative	Negative	Positive (5 days)	Positive
VPTB114B	Negative	Negative	Negative	Negative	VPTB117A	Negative	Negative	Positive (5 days)	Positive
VPTB115B	Positive (20 days)	Positive	Positive	Positive	-	-	-	-	-
VPTB116B	Positive (20 days)	Positive	Positive (5 days)	Positive	-	-	-	-	-
VPTB117B	Positive (20 days)	Positive	Positive (5 days)	Positive	-	-	-	-	-

PCR, polymerase chain reaction; TTD, time to detection.
